# Competition for IL-2 between Regulatory and Effector T Cells to Chisel Immune Responses

**DOI:** 10.3389/fimmu.2012.00268

**Published:** 2012-09-05

**Authors:** Thomas Höfer, Oleg Krichevsky, Grégoire Altan-Bonnet

**Affiliations:** ^1^Division of Theoretical Systems Biology, German Cancer Research CenterHeidelberg, Germany; ^2^Physics Department, Ben Gurion University of the NegevBeer-Sheva, Israel; ^3^Program in Computational Biology and Immunology, Memorial Sloan-Kettering Cancer CenterNew York, NY, USA

**Keywords:** computational modeling, cytokine competition, IL-2, regulatory T cells, systems immunology

## Abstract

In this review we discuss how the competition for cytokines between different cells of the immune system can shape the system wide immune response. We focus on interleukin-2 (IL-2) secretion by activated effector T cells (T_eff_) and on the competition for IL-2 consumption between T_eff_ and regulatory T cells (T_reg_). We discuss the evidence for the mechanism in which the depletion of IL-2 by T_reg_ cells would be sufficient to suppress an autoimmune response, yet not strong enough to prevent an immune response. We present quantitative estimations and summarize our modeling effort to show that the tug-of-war between T_reg_ and T_eff_ cells for IL-2 molecules can be won by T_reg_ cells in the case of weak activation of T_eff_ leading to the suppression of the immune response. Or, for strongly activated T_eff_ cells, it can be won by T_eff_ cells bringing about the activation of the whole adaptive immune system. Finally, we discuss some recent applications attempting to achieve clinical effects through the modulation of IL-2 consumption by T_reg_ compartment.

## Introduction

Recent studies, specifically on the role of IL-2 in the regulation of immune responses, have highlighted how cytokine competition may be a critical determinant to arbitrate the balance between tolerance and response, and/or to channel the activation of lymphocytes toward specific differentiation paths.

Cytokines are ubiquitous in immunology as mediators of cell–cell communications. Most knock-out mouse models of cytokines (with the notable exception of IFNγ) display critical and often deadly pathologies. For example, IL-2 knock-out mice are riddled with systemic autoimmune disorders (Horak et al., 1995), explained by the abrogated development and maintenance of regulatory T cells in peripheral lymphoid organs. On the other hand, there exist few clinical protocols whereby perturbations of cytokine pathways lead to clinical therapies. This review aims at presenting the need of quantitatively understanding cytokine function as a basis for more targeted therapeutic manipulation. We will discuss how the balance between cytokine secretion and consumption by multiple cell types fine-tunes the immune response.

This review is organized in four parts. First, we review recent experimental work addressing the role of cytokine consumption. Second, we present basic quantitative facts that highlight the explosiveness of cytokine secretion as well as the importance of cytokine consumption for lymphocyte–lymphocyte communication. We focus on IL-2 secretion and uptake as the best modeled case of cytokine competition, and also because it is a “self-contained” regulatory system (secreted by T cells, consumed by T cells). Third we summarize recent theoretical studies that addressed the role of IL-2 competition as a mechanism of suppression by regulatory T cells. Finally, we discuss the biological relevance of these theoretical efforts toward better understanding immunological regulations.

## Three Experimental Evidences Against a Role for IL-2 Depletion as a Mechanism for T_reg_ Suppression Can be Mitigated

The original identification of the CD4+CD25+ compartment as a key population to enforce peripheral tolerance (Sakaguchi et al., 1995) led to the conjecture that IL-2Rα played a critical role in T_reg_ function. Given the critical role of IL-2 for T cell survival and proliferation *in vitro*, many researchers originally conjectured that IL-2 depletion by T_reg_ cells would be a critical mechanism to enforce their suppressive capabilities. However, the autoimmunity observed in IL-2 knock-out mice drew into question an activating function for IL-2 for T cell immune responses *in vivo* (Kundig et al., 1993). A partial resolution of this perceived conundrum came from the observation that the development and maintenance of T_reg_ cells depends on IL-2, so that IL-2 was attributed an immunosuppressive – rather than an activating – function *in vivo*. However, careful studies that went beyond the constitutive IL-2 knock-out model have since demonstrated that the action of IL-2 on both CD4+CD25− and CD8+ T cells supports immune response in multiple ways (e.g., by sustaining different modes of proliferation of CD8+ T cells; Kundig et al., 1993; Williams et al., 2006; Cho et al., 2007), furthering the survival of CD4+ T cells (Dooms et al., 2004), and driving CD4 effector and memory cell differentiation (Yamane et al., 2005; Chen et al., 2011; Pandiyan et al., 2011). Thus IL-2 serves dichotomous functions in the suppression and enhancement of adaptive immunity.

While these studies open the possibility of an immunosuppressive role for IL-2 consumption by T_reg_ cells, experiments at the turn of the millenium have further challenged this idea. For example, experiments with a knock-out genetic model for the IL-2Rβ chain (CD122) delivered negative results regarding the role of IL-2 depletion by T_regs_ as a mechanism of suppression of autoimmune response. Specifically, Malek et al. (2002) relied on a thymic-transgenic expression of wild-type IL-2Rβ to drive the development of CD4+CD25+ T cells in the thymus then abrogate expression of IL-2Rβ in the periphery. These mice, whose peripheral lymphoid tissues contained CD4+IL-2Rα+IL-2Rβ− T_reg_ cells, were devoid of autoimmune disorders, while mice from straight IL-2Rβ−/− models lacked CD4+CD25+ cells and suffered systematic autoimmune attacks, analogously with IL-2Rα−/− models. This experimental observation was further analyzed and interpreted in terms of functional suppressive capabilities among IL-2Rβ-deficient T_reg_ cells, challenging the role of IL-2 signaling for T_reg_ suppression. However, more recent work mitigated this conclusion with the observation that peripheral T_reg_ cells from these IL-2Rβ thymic-transgenic knock-out mice retain their capabilities, albeit diminished, to respond to IL-2 (Bayer et al., 2007). These experimental inconsistencies may be explained by the recycling and long-term stability of the IL-2Rβ receptor, even after abrogation of its expression in the periphery. Hence, the accurate peripheral tolerance and lack of autoimmune disorders in IL-2Rβ thymic-transgenic knock-out mice can no longer be interpreted as a complete rejection of IL-2 depletion as a necessary mechanism for T_reg_ suppression.

The discovery of FoxP3 as the transcription factor that identifies unequivocally the T_reg_ lineage clarified the field, by offering a proprietary marker for T_reg_ that distinguishes them from transiently expressing CD4+CD25+ effector T cells. Work by the Rudensky lab (Fontenot et al., 2005) clearly established the critical role of IL-2 signaling for T_reg_ development and maintenance. On the other hand, this study showed that FoxP3+CD4+ cells from Il2ra−/− mice were as suppressive as T_reg_ from Il2ra-sufficient mice, at least in the classical *in vitro* proliferation assay: this observation (among others) again led to the conclusion that IL-2 signaling is dispensable for suppression. On the other hand, IL-2 signaling for Il2ra−/− FoxP3+ cells was not quantified and it is possible that compensatory mechanisms – e.g., upregulation of beta and gamma chains of the IL-2R receptors (Li et al., 2001) – would enable these IL-2Rα deficient cells to maintain their ability to respond and deplete IL-2. In particular, IL-2Rα−/− T cells have been shown to respond to IL-2, albeit at higher concentrations (1 nM instead of the characteristics 10 pM): this could explain why IL-2Rα−/− mice (that have FoxP3+ peripheral cells but at lower frequency than IL-2Rα sufficient mice) still suffer from systemic autoimmune disorders (a hallmark of defective suppression by T_reg_) but with less intensity than IL-2−/− mice (these mice are completely devoid of FoxP3+ cells).

A third line of experiments has previously been used to reject cytokine depletion as a mechanism for T_reg_ suppression and have led to the dogma that cell–cell contact between T_reg_ cells and T_eff_ cells is absolutely required for suppression. In the past, many groups (Shevach et al., 1998; Takahashi et al., 1998; Nakamura et al., 2001; Dieckmann et al., 2002; Xu et al., 2003) have used the classical transwell assay whereby T_reg_-T_eff_ contacts are forbidden by a membrane separation and found that this abolishes suppression of T_eff_ proliferation by T_reg_ cells. Unfortunately, this setup can potentially generate false-negative results – as originally suggested in Scheffold et al. (2005), Pandiyan et al. (2007). Indeed, as pointed out by Shevach (2009) in a recent review, “*It should be emphasized that the failure to observe suppression when T_reg_ cells are separated from the responder cells by a membrane does not rule out the possibility that T_reg_ cells secrete an as yet uncharacterized cytokine that functions in a gradient fashion and requires proximity between suppressor and responder*.” In this regard, it is noteworthy that the transwell geometry typically separate T_reg_ and T_eff_ cells by 4 mm when one uses the same Corning Costar transwell within a 24-well plate as described in Thornton and Shevach (1998). This is a very large distance to be bridged by diffusion. For IL-2 (a globular protein of 17 kDa), the coefficient of diffusion *D* in solution is of the order of 1 × 10^−6^ cm^2^/s (Weidemann et al., 2011). Thus the characteristic diffusion time *τ_D_* across *d* = 4 mm is: *τ_D_* = *d*^2^/(2*D*) = 40000 s = 11 h. Hence, the physical separation imposed by the transwell geometry implies a large time-delay between the secretion of soluble molecules by T_eff_ cells and their potential sensing (and scavenging) by T_reg_ cells (this time-delay might in fact be even larger, *τ*_mixing_ > 20 h, due to the low porosity of the transwell membrane). Under these conditions, paracrine and autocrine consumption of IL-2 by the T_eff_ population, rather than competitive take-up by the very distant T_reg_ cells will dominate (Scheffold et al., 2007; Busse et al., 2010; Feinerman et al., 2010). Thus abrogation of suppression in a T_eff_-T_reg_ transwell setup (Takahashi et al., 1998; Thornton and Shevach, 1998) does not rule out cytokine competition as one of the mechanisms of T_reg_ action (Scheffold et al., 2007). All in all, there is consensus in the field of regulatory T cells, regarding IL-2, that this cytokine is critical for the development and maintenance of this subpopulation. On the other hand, the three main lines of evidence dismiss the functional significance of IL-2 signaling in terms of the T_reg_ cells’ suppressive capacities may not be definitive.

## Functional Evidence for IL-2 Competition as One Mechanism for T_reg_ Suppression

The renaissance for IL-2 depletion as a mechanism for T_reg_ suppression came with studies from the Scheffold and Stockinger groups (de la Rosa et al., 2004; Barthlott et al., 2005; Brandenburg et al., 2008). Both groups documented how IL-2 depletion or blockage phenocopied the effect of T_reg_ cells on antigen-activated T cells. Conversely, either the exogenous addition of IL-2 or the blocking IL-2 uptake by T_reg_ cells only – and not by T_eff_ cells – was sufficient to abrogate suppression *in vitro*. Hence, IL-2 was conjectured to be a limiting factor for T_eff_ cell expansion *in vitro*. These groups then demonstrated that T_eff_ cells do produce IL-2 (despite reduced transcription of the IL-2 gene). An early predictor of suppressed T_eff_ cell expansion in these experiments was the lack of strong IL-2Rα expression on T_eff_ cells, accompanied in a reciprocal manner by further upregulation of IL-2Rα on T_reg_ cells. This behavior is readily explained by competitive IL-2 consumption through T_reg_ cells, as pSTAT5 drives IL-2Rα upregulation in both cell types. This reciprocal regulation of IL-2Rα in T_eff_ and T_reg_ cells has also been observed *in vivo* (Klein et al., 2003; Barthlott et al., 2005). Of note, IL-2 has been shown to prime T_reg_ cells for later expression of the immunosuppressive cytokine IL-10 *in vitro* and *in vivo* (Barthlott et al., 2005; Brandenburg et al., 2008).

These papers were followed by the comprehensive study by the Lenardo group (Pandiyan et al., 2007) that focused on the enhanced apoptosis among activated effector cells, when common-gamma chain (γ_c_) cytokines are missing because of their depletion by T_reg_ cells. All the hallmarks of cytokine deprivation-induced apoptosis (loss of phosphorylation of AKT, phosphorylation of BAD, membrane blebbing, resistance to death in Bim−/− mutants) were observed in the suppression assay *in vitro*. Moreover, Pandiyan et al. (2007) reported the measurements of reduced IL-2 concentration in supernatants of T_eff_-T_reg_ cocultures compared to T_eff_-only cultures: this was assigned to IL-2 consumption by T_reg_ cells rather than to reduced IL-2 production by T_eff_ cells. The measured IL-2 concentrations (around 1 unit/ml, i.e., 10 pM) were exactly in the range where maximal functional impact would be expected (see following section for details). Finally, a model of inflammatory bowel disease (IBD) was used as an *in vivo* assay of T_reg_ function: upon adoptive co-transfer of CD4+CD25+ T cells in SCID mouse, the onset of IBD was abrogated and colitogenic T_eff_ cells were shown to undergo apoptosis. Vice versa, when mice were not injected with CD4+CD25+ T cells, adoptively transferred CD45 T cells would proliferate and trigger IBD. Hence, Pandiyan et al. (2007) made a convincing case that depletion of IL-2 by T_reg_ cells constitute a critical mechanism to account for T_reg_ suppression.

## Quantitative Aspects of Cytokine Accumulation and Consumption

Addressing the role of cytokine depletion in enforcing suppression by T_reg_ cells depends on the quantitative understanding of the dynamics of cytokine accumulation and consumption in the extracellular medium of lymphoid organs. Of note, most cytokines are functional in concentration ranges (below 100 pM) that are unusual for most biological systems. Indeed, most ligand-receptor interactions, most hormones and growth factors operate in 10 nM to 10 μM range. Hence, there are specific challenges of the biophysics of cell–cell communication in the pM range that we need to address. In this section, we summarize the numbers related to IL-2 secretion and uptake, and estimate the kinetics of IL-2 accumulation in a lymph node. We note that the experimental uncertainties for kinetic rates and receptor numbers are rather large, so the correct parameter values might be within a factor of 2–3 from the stated value. In addition the receptor numbers and secretion rates are broadly distributed within the population and depend on the experimental protocols of measurement. Accordingly, we will keep our estimations simple, favoring clarity while aiming to stay within an order of magnitude of the actual parameter values.

First, we will briefly describe the kinetic steps involved in IL-2 signaling and consumption by T cells following the model presented in Feinerman et al. (2010). In general, IL-2 consumption by T cells proceeds in three steps (Feinerman et al., 2010):

1) Free IL-2 molecules reversibly bind α chains (IL-2Rα) of IL-2 receptors with characteristic on- and off-rates of *k*_weak_(+) = 1.4 10^7^/(M*s), *k*_weak_(−) = 0.4/s.
  $$\text{IL-2}+\text{IL-2R}\alpha ↔{\text{IL-2}}^{*}\text{IL-2R}\alpha$$
2) IL-2*IL-2Rα locks into a tight complex with available IL-2Rβ and γ_c_ chains of the IL-2 receptor with characteristic on- and off-rates of *k*_2_(+) = 3 × 10^−4^/s, *k*_2_(−) = 2.3 × 10^−4^/s, forming a complete IL-2*IL-2R receptor.
  $${\text{IL-2}}^{*}\text{IL-2R}\alpha +\text{IL-2R}\beta^{*}\gamma_{c}↔{\text{IL-2}}^{*}\text{IL-R}$$
  The assembly of the tetrameric cytokine/receptor complex triggers the phosphorylation of the transcription factor STAT5 into pSTAT5. pSTAT5 molecules dimerize and enter the cell nucleus where they regulate a variety of genes, among them many genes associated with cell survival and proliferation. Importantly within the context of IL-2 communication, pSTAT5 upregulates the expression of IL-2Rα and downregulates the secretion of IL-2.
3) IL-2*IL-R complex is internalized by the cells with the rate of *k*_endocytosis_ = 1.1 × 10^−3^/s = (15 min)^−1^ (Duprez and Dautry-Varsat, 1986; Duprez et al., 1988, 1991; Hemar et al., 1995). We assume further that upon internalization the IL-2R receptor chains return to the cell membrane. This constitutes a simple way to model the conditions at the cell membrane as quasi-stationary. In experiments, the quasi-stationary conditions are supported by the fact that cytokine consumption dynamics happens at the time scales of several minutes, while the numbers of receptor chains remain stable over the course of several hours.

For the kinetic rates and numbers of receptors chains typical for T cells, the two-step model for IL-2 binding and uptake gives the number *N*_R_ of assembled IL-2*IL-R complexes per cells as:

(1) $$N_{R}\approx \frac{N_{\beta \gamma }}{1+\frac{K}{N_{\alpha }\left[IL-2\right]}}$$

and the IL-2 consumption rate by the cell of

(2) $$J_{\text{cons}}=k_{\text{endocytosis}}N_{\text{R}}\approx \frac{k_{\text{endocytosis}}N_{\beta \gamma }}{1+\frac{K}{N_{\alpha }\left[IL-2\right]}}$$

where $K=\frac{\left(k_{2\left(-\right)}+k_{\text{endocytosis}}\right)k_{\text{weak}\left(-\right)}}{k_{\text{weak}\left(+\right)}k_{2\left(+\right)}}$ and *N*_α_, *N*_βγ_ are the numbers of IL-2Rα and of IL-2Rβ*γ_c_ complexes per cell respectively.

The first main consequence of this model is that the EC50 for IL-2 signaling (defined as the concentration of IL-2 that yields 50% of the pSTAT5 response) is inversely proportional to the number of IL-2Rα chains per cell. In particular, for a moderately activated T cell with *N*_α_ ≈ 10^4^, EC50 ∼10 pM, while for a strongly activated T cell with *N*_α_ ≈ 10^5^, EC50 ∼1 pM. The second consequence of the model is that at high IL-2 concentrations ([IL-2] ≫ EC50), IL-2 signaling and consumption reach saturation and are limited by the number of IL-2Rβ*γ_c_ complexes that has been estimated to be ∼300 in the naïve T cell and rises to ∼1000 upon activation (Feinerman et al., 2010). In general, this number appears to be limited by the number of available γ_c_ chains: although there typically ∼5000–8000 of them per cell, they are shared between many types of different cytokine receptors: the best estimates for the number of available γ_c_ came from fitting IL-2 consumption by T_reg_ cells (Feinerman et al., 2010). Notice that the consumption rate being rather low *k*_endocytosis_ = 1.1 × 10^−3^/s, a strongly activated cell exposed to saturating concentrations of IL-2 would be able to consume at most one molecule of IL-2/s.

IL-2 secretion starts only upon activation of naïve T cells with foreign antigens. Within hours of activation through their TCR signaling pathways, effector T cells start secreting IL-2 at an average rate of ∼10 molecules/s (our unpublished data). As IL-2 is secreted in the extracellular medium, it diffuses away from the secreting cell and the IL-2 field around the cell is established with the maximal concentration near the cell with a characteristic decay length of about cell radius R. The IL-2 concentration near the cell c_0_ is set by the balance between IL-2 secretion and its diffusion:

$$\begin{array}{cccc} J_{\sec} & =4\pi R^{2}D\nabla c\approx 4\pi R^{2}D\frac{c_{0}}{R} & & \\ c_{0} & \approx \frac{J_{\sec}}{4\pi RD} & & \text{(3)} \end{array}$$

where *D* is the diffusion coefficient for IL-2. Its value in an aqueous buffer is ∼100 μm^2^/s, but the diffusion coefficient within a lymph node is not known. Assuming that the viscosity of the extracellular matrix is similar to that of the cell cytoplasm (∼6 times higher than that of aqueous solution), we could estimate *D* ∼ 16 μm^2^/s. Then using R ≈ 5 μm, we arrive at *c*_0_ ≈ 20 pM, which is larger than EC50 of even moderately activated T cells. Thus an activated secreting cell should be able to sense IL-2 it produces in an autocrine manner. However, at the onset of activation T cells lack IL-2Rα chain and would need concentrations of IL-2 larger than 1 nM to respond to it. The expression of IL-2Rα is initiated upon cell activation, and within 24–48 h the number of IL-2Rα reaches ∼10^4^–10^5^ per cell lowering EC50 to ∼1–10 pM of IL-2 and allowing for cell sensing its own field of IL-2.

However, even a strongly activated cell would consume only a small fraction of secreted IL-2 in an autocrine matter. Indeed, as discussed above a strongly activated T cell can consume at most ∼1 IL-2 molecule/s while producing 10 molecules/s on average. The rest of the molecules would in principle accumulate within the lymph node and contribute to communication between different T cells and to coordination and strengthening of the system immune response. Assuming there are ∼200 activated T cells in a draining lymph node ∼48 h after immunization (there are typically few hundreds T cells in a body tuned to activate to a particular peptide, not all of them will reach the lymph node and those activated in the node will experience 1–2 divisions during that time) and taking the volume of a lymph node to be 1 μl, we estimate that the IL-2 concentration in the node would reach 48 h × (3600 s/h) × (10 molecules/s/cell) × (200 cells)/1 μl/*N*_Avogadro_ ∼ 600 pM within 48 h of secretion. Such IL-2 concentration should indeed allow for strong signaling and cross communication between activated T cells. Specifically, even weakly activated cells (i.e., cells with lower levels of IL-2Rα would be able to phosphorylate STAT5: cell–cell communication through IL-2 would be rather unspecific and universal for all T cells in a lymphoid organ.

However, such accumulation of IL-2 is prevented by regulatory T cells. Unlike naïve T cells, T_reg_ cells express constitutively all of the chains of IL-2R even in homeostasis (∼10^4^ of IL-2Rα and ∼300 of IL-2Rβ*γ_c_) and can consume IL-2 from the onset of an immune response. Thus concentration of IL-2 will be established by the balance of overall secretion rate by T_eff_ cells and the consumption rate by T_reg_ cells. There are ∼5 10^6^ T cells in a lymph node and ∼5% among them are T_reg_ cells, i.e., ∼2.5 10^5^ cells. Comparing their consumption rate from Eq. 2 to the secretion rate of 200 activated T cells (i.e., 2000 molecules/s), we estimate that in first 24–48 h, IL-2 concentration does not exceed 0.15 pM. This level is too low for signaling to occur, so there will be almost no cross talk between different T_eff_ cells. Similarly, with the exception of cells in the immediate vicinity of a secreting T_eff_ cell, the majority of T_reg_ cells do not activate their pSTAT5 and downstream genes.

For an efficient cross talk to occur, the IL-2 concentration has to rise about tenfold to ∼1 pM, which is achieved when the number of activated T cells reaches ∼2000, which could take another 24–48 h. Well activated T_eff_ cells (with EC50 ∼1 pM) signal much more efficiently than the majority of T_regs_ (EC50 ∼10 pM) at these IL-2 concentrations. As described above, IL-2 signaling leads to upregulation of IL-2Rα expression and therefore to yet stronger signaling by T_eff_ cells allowing them from that stage onward to win over T_regs_ in the competition for IL-2. Only a minority of T_reg_ cells, those in the immediate vicinity of a secreting cell, have chances to keep up with T_eff_ cells. The number of such T_reg_ cells can be estimated from the probabilistic argument: since in a close packed situation each cell has ∼12 neighbors and 5% of those are T_regs_, the number of strongly signaling T_reg_ cells should be ∼60% of activated T helper cells. However, as T_eff_ further proliferate the importance of IL-2 consumption by T_regs_ becomes negligible (Sojka et al., 2005, 2008). IL-2 secreted by the weakly activated (e.g., autoimmune) T_eff_ cells would be consumed mostly by T_reg_ cells; the T_eff_ cells would not be able to cross-communicate and their response would be suppressed. Yet T_eff_ cells strongly activated by the foreign proteins will be able to eventually overcome the suppression, and to exchange IL-2 cytokines and coordinate their response.

In addition to the competition between T_eff_ and T_reg_ cells, there exists competition for IL-2 within the T_eff_ system. As noted above, IL-2 signaling pathway leads to both upregulation of the expression of IL-2Rα and downregulation of IL-2 secretion. This means that T_eff_ cells that got more IL-2Rα signal stronger than others, which leads them to express more IL-2Rα and achieve yet stronger signaling capabilities. At the same time, they produce less and less of IL-2. Thus these two feedbacks are two mechanisms that bring about the split of T_eff_ population into two subpopulations: “consumers” and “producers.” “Consumers” have shut down their IL-2 secretion, but have plenty of IL-2Rα that allows them to “steal” IL-2 from “producers.” The “producers” are stuck in IL-2 secretion state since they have few IL-2Rα and so little chances of starting signaling and changing their state as their IL-2 is being stolen by the “consumers.” This consideration implies that a continuum of IL-2Rα levels within the T_eff_ cell population may yields to a digital split between IL-2 consumers and producers, with functional consequence in terms of differentiation (effector vs. memory phenotypes). Note that the heterogeneity between producers and consumers is not exclusively due to negative feedback in IL-2 secretion, as production is heterogeneous from the outset (Hollander et al., 1998; Podtschaske et al., 2007). This has been particularly well studied in the context of other cytokines (Mariani et al., 2010), but results to be published will confirm this endogenous variability in IL-2 production. Ultimately, competition for cytokines and heterogeneity in T cell signaling are critical contributors for T cell diversification during differentiation. For example, Catron et al. (2006) found that antigen-specific CD4+ cells arriving late into the draining lymph node divide less and their progeny is more likely to become memory cells than that of resident cells.

## Modeling the IL-2 tug-of-war between T_reg_ and T_eff_ Cells: A Systems Immunology Challenge

A conspicuous feature of IL-2 and other T cell cytokines is their action as both autocrine and paracrine messengers. Thus a key question in understanding the physiological effects of cytokines is: which cells actually receive the cytokine signal? Does the secreting cell consume most of it in an autocrine manner, or is it quickly distributed to neighboring cells – and if so, how far does the signal travel in space? Moreover, cytokine signaling is likely to be highly dynamic in time and space since cytokines regulate the expression of genes involved in the signaling – most prominently cytokine genes themselves as well as cytokine receptor genes.

To understand how the rates of cytokine production, diffusion, and cellular consumption as well as the feedback regulation of cytokine signaling shape the biological action of a cytokine, mathematical models provide an appropriate tool. Such models incorporate in a systematic way the kind of arguments made in the previous section, in order to simulate the dynamics of cytokine signaling for populations of secreting consuming and signaling cells. Two recent studies have employed a combination of experiments and mathematical modeling to dissect IL-2 signaling between antigen-activated, IL-2 secreting CD4 T cells and regulatory T cells (Busse et al., 2010; Feinerman et al., 2010). These studies provide mechanistic insight into how a single cytokine can serve as a messenger to two opposing T cell subsets.

**Figure 1:**
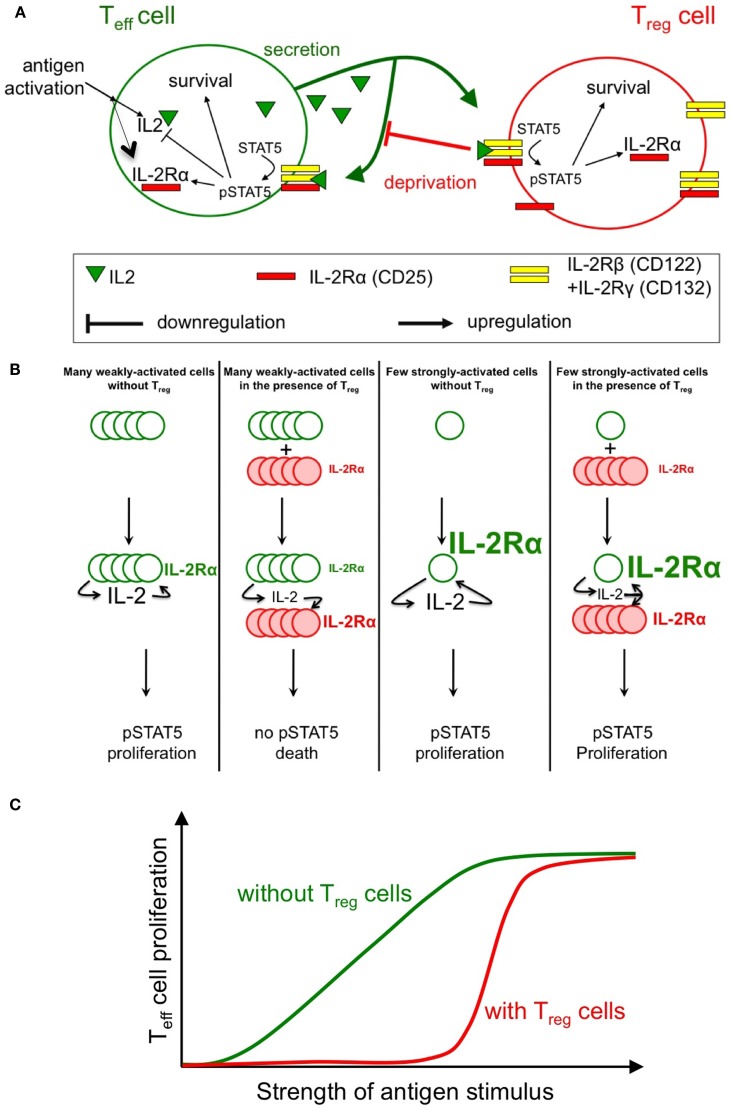
**Sketch of the feedback involved in the regulation of IL-2 response**. Antigenic engagement of T_eff_ cells leads to their activation, with IL-2Rα upregulation and IL-2 production **(A)**. Studies by our groups (Busse et al., 2010; Feinerman et al., 2010) quantified the strength of feedback regulations on IL-2 signaling and secretion. In particular, the role of T_reg_ cells (whose constitutive expression of IL-2Rα allows early IL-2 depletion) in regulating STAT5 phosphorylation in T_eff_ cells was modeled *in silico*. Three main predictions from our models were validated experimentally: (1) T_reg_ can impact a double suppressive hits on T_eff_ cells by blocking IL-2Rα upregulation and IL-2 accumulation hence abrogating STAT5 phosphorylation **(B)**; (2) This suppression is highly dynamic and variable: in particular, T_reg_ cells can rely on the competition for IL-2 to block the proliferation of weakly activated T_eff_ cells, while allowing strongly activated T_eff_ cells to mount an immune response **(C)**; note how the presence of T_reg_ cells reduce the overall proliferation of T_eff_ cells but, as well, sharpen the dose responsive curve for proliferation vs. antigen strength; (3) Complex spatio-temporal coupling allow T_reg_ cells to modulate and regulate the extent of suppression in crowded environments (e.g., *in vivo*).

Our two theoretical models describe the binding of IL-2 to IL-2 receptors, downstream signal transduction via the Stat5 pathway and activation of IL-2Rα gene expression (Figure 1A). Feinerman et al. (2010) provide a detailed description of signal transduction and its cell-to-cell heterogeneity and also include feedback repression of the IL-2 gene. Busse et al. (2010) study the spatial aspect of signaling in dense cell assemblies that is governed by the interplay between IL-2 diffusion and competitive uptake. Various experimental measurements were used by our two groups to parameterize these models and make testable predictions.

Both quantitative models consistently show that there exist three very different biological outcomes of IL-2 secretion by antigen-stimulated responder T cells, depending on two parameters: (1) the rate of IL-2 secretion and (2) the presence (or absence) of proximal T_reg_ cells. In the absence of T_reg_ cells, even weak antigenic stimulation of responder T cells and, correspondingly, low IL-2 secretion rates are sufficient to upregulate expression of IL-2Rα and trigger the IL-2/Stat5 pathway in the responder T cells. By contrast, in the presence of T_reg_ cells that constitutively express IL-2Rα, the T_reg_ cells will completely deprive responder T cells of their own IL-2 and prevent Stat5 activation. Thus, with a stimulation at low concentration of antigens, the outcome of IL-2 signaling is dichotomic: either the responder T cells receive the signal (no T_reg_ cells present) or it is completely consumed by the T_reg_ cells (Figure 1B). IL-2 deprivation by T_reg_ cells will therefore quench spurious activation events and sharpen the antigen activation threshold (Figure 1C). However, strongly antigen-stimulated responder T cells will produce sufficient IL-2 so that both the responder T cells and nearby T_regs_ will share the signal. As IL-2 primes T_reg_ cells for later IL-10 expression *in vivo* (Brandenburg et al., 2008), this sharing of IL-2 can both support an immune response and initiate a delayed negative feedback loop (Scheffold et al., 2005; Yamaguchi et al., 2011). Taken together, these findings provide a mechanistic underpinning for IL-2 competition as a suppressive mode of T_reg_ cells that depends on the strength of the antigen stimulus (de la Rosa et al., 2004; Barthlott et al., 2005; Pandiyan et al., 2007; McNally et al., 2011).

The positive feedback regulation of IL-2Rα chain expression by IL-2/Stat5 signaling plays a critical role in this regulatory network. In responder T cells, full IL-2Rα expression and formation of high-affinity IL-2 receptors requires, in addition to the antigen stimulus, phosphorylation of Stat5. Hence T_reg_ cells, which constitutively express IL-2Rα, will deprive of IL-2 weakly stimulated responder T cells (here, cells that fail to upregulate IL-2Rα sufficiently) and keep them from expressing high-affinity IL-2 receptors, thus inflicting a “double hit” (Feinerman et al., 2010). However, if IL-2 is abundant (strongly stimulated) responder T cells upregulate their own IL-2Rα expression due to positive feedback. These activated cells do no longer suffer from IL-2 deprivation by T_regs_ because they have themselves become efficient sensors and consumers of the cytokine. Under conditions where IL-2Rα is limiting for the formation of high-affinity IL-2 receptors, the IL-2–IL-2Rα positive feedback can function as a digital switch that converts graded changes in the antigen stimulus into an all-or-nothing decision for cell proliferation at the single-cell level (Busse et al., 2010). Quantitation of the IL-2R subunit expression and resulting Stat5 phosphorylation in responder T cells shows that both IL-2Rα and IL-2Rβ levels control the responsiveness of a cell to IL-2. Moreover, cell-to-cell variability in the expression of both receptor subunits results in a broad distribution of IL-2 sensitivities in a cell population (Feinerman et al., 2010).

Interestingly, a recent systems-biology study of an unrelated cytokine pathway, Epo signaling in erythroid progenitors, has shown a key role for signal processing of cytokine consumption by rapid receptor turnover (Becker et al., 2010). However, what makes the IL-2–IL-2R system ideally suited for cytokine competition is the positive feedback regulation of receptor (IL-2Rα) expression in both responder T cells and T_reg_ cells. Through this self-amplification of IL-2 signaling and consumption, rather subtle initial differences in strength and timing of antigen stimulation can lead to clear-cut biological outcomes (Feinerman et al., 2010).

An important question that has already been introduced in the discussion of the transwell assay is how far a cytokine signal can travel in space – Section “*Three Experimental Evidences Against a Role for IL-2 Depletion as a Mechanism for T_reg_ Suppression can be Mitigated*.” Clearly, diffusion over mm-range distances, with diffusion times of several hours (as in the transwell setup), is prohibitive for efficient communication. However, a biologically more relevant question is whether cytokine gradients occur on a smaller scale that could compartmentalize cell-to-cell signaling in lymphoid organs. For *in vitro* experiments, Feinerman et al. (2010) estimated that diffusion through the supernatant would not allow steep concentration gradients to develop (and moreover, convection, which is much faster than diffusion over larger distances, is also likely to occur in typical *in vitro* setups). By contrast, the explicit modeling of diffusion in rather dense cell assemblies (where extracellular space and total cell volume are of comparable magnitude) show that competitive IL-2 uptake under conditions of limited supply can cause strong concentration gradients (Busse et al., 2010). This is particularly evident for T_reg_ cells, which due to their high constitutive IL2-Rα expression function as potent sinks of the cytokine. As a consequence, T_reg_ cells can absorb the IL-2 secreted by localized, weakly stimulated responder T cells and thus prevent the paracrine spread of this signal to other responder T cells in the neighborhood. On the other hand, when IL-2 secretion is strong (owing to a high fraction of secreting responder T cells and/or high secretion rates), T_reg_ cells will become saturated and the IL-2 signal could pervade an entire lymph node [as shown previously for IL-4 (Perona-Wright et al., 2010)]. In summary, the modeling suggests that T_reg_ cells also control the spatial propagation of IL-2 signals in lymphoid organs.

By iterating between modeling and experiments, the studies by Busse et al. (2010) and Feinerman et al. (2010) have revealed an unexpected plasticity of the IL-2 cytokine network, where quantitative parameters (secretion rate, diffusion, and competitive uptake) shape the biological outcome. Further theoretical studies have modeled T cell population dynamics and IL-2 signaling (Burroughs et al., 2006), making experimentally testable predictions. As several other cytokines of the adaptive immunity share principle features of the IL-2 system, especially competitive uptake by different cell populations and feedback regulation of signaling, we expect that similar “behavioral” plasticity will be found also in other cytokine networks.

## Perturbing the IL-2 tug-of-war to Maximize Immunotherapeutic Impact

At the same time as the issue of cytokine competition was being revisited, pre-clinical and clinical studies have put forward the possibility of applying IL-2 treatments to manipulate the T_reg_ compartment and impact on clinical outcomes (Murphy et al., 2012). Specifically, using antibody to cross-link IL-2 and to increase its lifetime *in vivo* (bare IL-2, because of its low molecular weight, gets filtered out of the system, mostly in the kidneys), researchers discovered that the T_reg_ compartment could be expanded. For example, Boyman and colleagues achieved proliferation of CD4+FoxP3+ lymphocytes in mice, increasing the frequency of T_reg_ cells by 10-fold, 3 days post-injection of the cytokine/antibody complex (Webster et al., 2009). The functional significance of this observation was immediately tested on a model of experimental autoimmune encephalomyelitis (EAE) whose induction could be abrogated after such robust T_reg_ cell expansion.

Similar observations (whereby the T_reg_ compartment is expanded with high levels of IL-2Rα and enhanced suppressive capacities) have been reported in other models. The Bluestone group applied a low-dose regiment of IL-2 to NOD mice, and obtained a strong delay or complete abrogation of diabetes (Tang et al., 2008): concomitantly, they reported that T_reg_ cells harvested from the pancreatic islet were more abundant (27% instead of 7% among CD4+ T cells) and expressed higher levels of IL-2Rα (20-fold higher). Similar results were obtained in a clinical setting, whereby low-dose regimen of IL-2 was demonstrated to be sufficient to delay the onset of Graft-vs.-Host-disease in allogeneic bone marrow transplant settings (Koreth et al., 2011). Again, these results should be analyzed quantitatively to test whether the subtle balance between immune response (autoimmune attack of the pancreatic islet or allogeneic activation of graft T cells) and immune tolerance (suppression of activation and proliferation) could result from the boosted ability of T_reg_ cells to compete for cytokines. A model for such immunotherapeutic intervention has already been proposed and makes further experimental testing critical. IL-2 regimen clearly upregulates IL-2Rα levels (with enhanced ability to bind and deplete IL-2), but it could also trigger other suppression mechanisms (e.g., secretion of IL-10 or upregulation of CTLA4).

Further quantitative modeling of this cytokine competition within clinical settings will thus be necessary to test and optimize cytokine competition, mostly to block autoimmune disorders using natural suppressive capabilities, but also to boost cytotoxic impact in cancer immunotherapies (2012). Although, this may prove difficult as direct and repeated probing of the tissue of interest (skin in GvHD, pancreatic islets in diabetes), we conjecture that mathematical models will become more and more critical to interpret functional changes as measured among accessible peripheral blood mononuclear cell (e.g., IL-2Rα upregulation in an expanded T_reg_ compartment) and extrapolate them to the tissue of relevance. Note that accurate measurements of *in vivo* concentrations of cytokines would go a long way toward resolving issues about cytokine communications in the immune system: this remains a challenging task given the low levels of free cytokine within tissues, but technical developments (ELISA miniaturization and the use of physiological reporters for cytokines) will solve this problem in the coming years. Ultimately, mouse pre-clinical models will be particularly useful to fine-tune the blood-to-tissue quantitative interpolation of immune responses.

## Conclusion

We presented a review of recent efforts in Systems Immunology that aim at addressing the role of cytokine competition as one mechanism of immune suppression by T_reg_ cells. Modeling quantitatively how cytokine is secreted by effector T cells undergoing activation, and how much gets scavenged by regulatory T cells remains challenging because of the dynamic complexity of the system. However, computational models have already highlighted the spatio-temporal intricacies of IL-2 competition: depending on the speed of IL-2Rα upregulation among T_eff_ cells, there exists a time window when T_reg_ cells deplete the extracellular medium of the secreted IL-2 and “snuff” this critical cytokine for differentiation. Spatially, the tight-packed space of lymphoid organs as well as the high density of polyclonal T_reg_ cells with high tonic level of IL-2Rα expression can limit the spatial extent at which T_eff_ cells can communicate through IL-2 sharing. This in turns, can act as a differential regulatory mechanisms to discriminate between activation of T_eff_ cells with low or high concentrations of antigens. Future work will need to extend the modeling framework introduced for IL-2 to other cytokines (e.g., IL-4, IL-10, TGFβ) as well as other costimulatory signals (e.g., B7/CTLA4). Further modeling effort will need to deal with cell proliferation and homeostasis, as proposed in a recent study (Burroughs et al., 2011; Almeida et al., 2012). These quantitative approaches will contribute greatly to assess the relevance of these varied mechanisms of T_reg_ cell suppression *in vitro* and *in vivo*. Beyond fundamental immunology, such quantitative insight may open new avenues of cytokine perturbation, to maximize immunotherapeutic impact in clinical settings.

## Conflict of Interest Statement

The authors declare that the research was conducted in the absence of any commercial or financial relationships that could be construed as a potential conflict of interest.
